# Trends in US Preterm Birth Rates by Household Income and Race and Ethnicity

**DOI:** 10.1001/jamanetworkopen.2025.50664

**Published:** 2026-01-02

**Authors:** Erika G. Cordova-Ramos, Stacey Y. Ruiz, Genevieve G. Guyol, Nikita S. Kalluri, Mei Elansary, Margaret McConnell, Margaret G. Parker

**Affiliations:** 1 Department of Pediatrics, Boston Medical Center, Boston, Massachusetts; 2 Evans Center for Implementation and Improvement Science, Boston, Massachusetts; 3 Department of Pediatrics, Boston University Chobanian and Avedisian School of Medicine, Roxbury, Massachusetts; 4 Department of Pediatrics, University of Massachusetts Chan Medical School, Worcester; 5 Department of Global Health and Population, Harvard T.H. Chan School of Public Health, Boston, Massachusetts

## Abstract

**Importance:**

There are well-documented racial and ethnic disparities in preterm birth in the US. The role of household income in temporal preterm birth trends remains largely unexplored.

**Objective:**

To examine US preterm birth trends by household income from 2011 to 2021, as well as associations between income and preterm birth according to race and ethnicity.

**Design, Setting, and Participants:**

This US population-based, cross-sectional study used data on 411 469 mothers of infants aged 2 to 4 months from the nationally representative Pregnancy Risk Assessment Monitoring System database from 2011 to 2021. Data were analyzed from January to April 2024.

**Exposures:**

Mothers reported annual household income, which was categorized as less than 100% of the federal poverty level (FPL), 100% to 199% of the FPL, and 200% or more of the FPL based on year, state, and household size.

**Main Outcome and Measures:**

The main outcome was preterm birth, defined as birth at less than 37 weeks’ gestation. Maternal self-reported race and ethnicity was defined as American Indian or Alaska Native, Asian, Hispanic (any race), non-Hispanic Black, non-Hispanic White, and other or multiracial. Trends in preterm birth by income categories were examined, and modified Poisson regression models were built to (1) examine the association between income and preterm birth, (2) adjust for sociodemographic and pregnancy-related covariates, (3) adjust additionally for race and ethnicity, and (4) introduce an interaction between race and ethnicity and income.

**Results:**

Among 411 469 (weighted 20 million) mother-infant dyads (0.8% American Indian or Alaska Native, 5.5% Asian, 15.5% Hispanic, 14.1% non-Hispanic Black, 58.9% non-Hispanic White, and 3.1% other or multiracial), rates of preterm birth increased significantly over time in the groups reporting an annual household income of less than 100% of the FPL (2011, 9.7%; 2021, 11.1%) and 100% to 199% of the FPL (2011, 7.8%; 2021, 10.0%). The preterm birth rate was highest among households reporting household income less than 100% of the FPL within all racial and ethnic groups except Asian. Non-Hispanic Black mothers had the highest rates of preterm birth across all income categories. The association of income with preterm birth remained significant after adjusting for covariates but attenuated to the null after including race and ethnicity in the model. In the lowest income group, non-Hispanic Black mothers had a 19% greater risk of preterm birth compared with non-Hispanic White mothers (adjusted relative risk [ARR], 1.19; 95% CI, 1.11-1.27), whereas in the highest income group, non-Hispanic Black mothers had a 13% greater risk of preterm birth compared with non-Hispanic White mothers (ARR, 1.13; 95% CI, 1.01-1.26).

**Conclusions:**

In this population-based cross-sectional study, household income disparities in preterm birth widened over time. Black race moderated the association between income and preterm birth, underscoring the need to examine the role of racism in preterm birth disparities.

## Key Points

**Question:**

How have US preterm birth trends varied by household income over time, and to what extent are race and ethnicity associated with this variance?

**Findings:**

In this cross-sectional study of 411 469 births using 2011-2021 Pregnancy Risk Assessment Monitoring System data, preterm birth rates increased among low-income households (<200% of the federal poverty level) but remained stable among higher-income households. Race moderated the association between income and preterm birth; Black mothers had the highest preterm birth rates across all income levels.

**Meaning:**

In this study, racial and ethnic disparities in the preterm birth rate persisted, suggesting that interventions addressing income alone may be insufficient to reduce inequities in preterm birth.

## Teaser

This cross-sectional study uses nationally representative data from the Pregnancy Risk Assessment Monitoring System database to examine temporal US preterm birth trends by income categories from 2011 to 2021, as well as associations between income and preterm birth according to race and ethnicity.

## Introduction

Preterm birth, defined as delivery before 37 weeks’ gestation, remains the leading cause of infant mortality and morbidity in the US.^1^ Among US populations, non-Hispanic Black individuals have approximately twice the risk of preterm birth compared with non-Hispanic White individuals, a disparity that has remained unchanged for decades.^2^ Household income, which often intersects with race and ethnicity, may be associated with risk of preterm birth in several ways. Poverty influences neighborhood environments, access to nutritious food and high-quality health care, exposure to pollutants, and the burden of chronic conditions and chronic stress, all of which can be directly or indirectly associated with gestational outcomes.^3^ Although income is a key social factor associated with health, national trends of preterm birth by household income, and the extent to which income-based patterns differ within racial and ethnic groups, remain largely unexplored. To fill this gap, we used nationally representative data to examine temporal trends in preterm birth by income categories and the extent to which associations between income and preterm birth differ across racial and ethnic populations in the US.

## Methods

### Data Source

We used data from the Pregnancy Risk Assessment Monitoring System (PRAMS), a cross-sectional, multistate perinatal surveillance initiative developed by the Centers for Disease Control and Prevention (CDC) and administered by state health departments. PRAMS surveys mothers 2 to 4 months post partum to collect detailed information on perinatal experiences, health behaviors, and outcomes. These survey responses are then linked with birth certificate data, which provide additional information, including maternal demographic characteristics (eg, age and race and ethnicity), pregnancy characteristics (eg, parity and plurality), and infant outcomes (eg, gestational age and birth weight).^4^ The system uses stratified, weighted sampling so that estimates are representative at the population level, particularly for historically marginalized groups. Further details on PRAMS methods have been previously published.^5^ The institutional review board of Boston Medical Center and Boston University Medical Campus determined that PRAMS data use was in the exempt category of research because it is a secondary analysis of deidentified survey data; thus, informed consent was not required. This study followed the Strengthening the Reporting of Observational Studies in Epidemiology (STROBE) reporting guideline for cross-sectional studies.^6^

### Study Design and Population

We conducted a population-based, cross-sectional study using PRAMS data from 2011 to 2021. We excluded mother-infant dyads with missing information on completed weeks of gestation (n = 664), race and ethnicity (n = 16 408), or household income (n = 46 318), resulting in a final unweighted sample of 411 469 mothers for analysis (weighted 20 million).

### Measures

Gestational age was obtained from birth certificate data. Household income was reported by mothers in categorical ranges. To minimize misclassification of households into lower-income groups, we conservatively estimated income status by assuming the upper value of a woman’s income band and converted it to a percentage of the federal poverty level (FPL) based on the corresponding year, state, and household size.^7^ We categorized income as less than 100% of the FPL, 100% to 199% of the FPL, and 200% or more of the FPL. Maternal self-reported race and ethnicity was obtained from birth certificate data and categorized as American Indian or Alaska Native, Asian, Hispanic (any race), non-Hispanic Black (hereafter, *Black*), non-Hispanic White (hereafter, *White*), and other race or multiracial (specific races and ethnicities not specified). Covariates included sociodemographic and pregnancy-related factors known to be associated with income and/or preterm birth, such as maternal age, educational level, language, prepregnancy insurance status (private, public [Medicaid], or none), prenatal care (Kessner Index),^8^ diabetes (before or during pregnancy), hypertension during pregnancy, history of preterm birth, smoking during pregnancy, and multiple births.

### Statistical Analysis

Data were analyzed from January to April 2024. We applied survey-specific procedures to incorporate the sampling weights, strata, and cluster variables provided by the CDC. This approach ensured appropriate variance estimation and accounted for the complex survey design of the PRAMS dataset.

We assessed annual preterm birth prevalence by income category and used the Cochran-Armitage test to evaluate trends over time.^9^ To explore whether preterm birth patterns differed by race and ethnicity, we first stratified preterm birth rates by racial and ethnic group within each income level. We then constructed a series of modified Poisson regression models: (1) unadjusted, (2) adjusted for sociodemographic and pregnancy-related factors, (3) additionally adjusted for race and ethnicity, and (4) additionally including an interaction term between race and ethnicity and income. We used modified Poisson regression with a log link and robust standard errors to directly estimate relative risks.^10^

Because 10.8% of observations were missing data on household income, we conducted 2 sensitivity analyses consistent with recommendations for population-based surveys.^11^ First, we compared key characteristics of participants with complete vs missing income data (eTable 1 in Supplement 1). For the first sensitivity analysis, we retained missing-income cases by adding a “missing income” category and reestimated all analyses including this category (eTables 2-4 in Supplement 1). For the second sensitivity analysis, we performed multiple imputation using the fully conditional specification method and reestimated all analyses (eTables 5 and 6 in Supplement 1). Variables used for imputation were maternal age, race and ethnicity, educational level, marital status, and health insurance. All hypothesis tests were 2-sided, with statistical significance set at *P* < .05. Data were analyzed using Stata, version 14.0 (StataCorp LLC).

## Results

We analyzed 411 469 mother-infant dyads participating in PRAMS between 2011 and 2021, representing a weighted population of 20 million (0.8% American Indian or Alaska Native, 5.5% Asian, 15.5% Hispanic, 14.1% non-Hispanic Black, 58.9% non-Hispanic White, and 3.1% other or multiracial). Overall preterm birth rates across our study period were 10.4% for mothers with household incomes less than 100% of the FPL, 8.9% for mothers with household incomes 100% to 199% of the FPL, and 7.5% for mothers with household incomes 200% or more of the FPL. Demographic characteristics varied markedly by household income level, with lower-income groups more likely to be younger, members of racial and ethnic minority populations, Spanish speaking, and publicly insured (Table 1).

**Table 1.** Baseline Characteristics of Mother-Infant Dyads in the PRAMS Study Population, 2011-2021 (N = 411 469; Weighted 20 Million)

Characteristic	Total No. (%)	No. (%) stratified by FPL		
		<100% (n = 4 593 438)	100%-199% (n = 3 905 488)	≥200% (n = 9 176 385)
Maternal age, y				
≤19	1 011 263 (5.0)	485 796 (10.6)	173 902 (4.5)	53 467 (0.6)
20-34	15 505 816 (77.4)	3 600 038 (78.4)	3 202 526 (82.0)	6 963 393 (75.9)
≥35	3 529 608 (17.6)	507 526 (11.1)	528 961 (13.5)	2 159 346 (23.5)
Race and ethnicity				
American Indian or Alaska Native	166 355 (0.8)	77 544 (1.7)	39 115 (1.0)	30 507 (0.3)
Asian	1 100 827 (5.5)	166 285 (3.6)	180 282 (4.6)	611 249 (6.7)
Hispanic	3 108 217 (15.5)	1 107 133 (24.1)	750 334 (19.2)	652 286 (7.1)
Non-Hispanic Black	2 830 208 (14.1)	1 067 206 (23.2)	666 554 (17.1)	586 484 (6.4)
Non-Hispanic White	11 797 803 (58.9)	1 894 702 (41.3)	2 039 967 (52.2)	6 904 253 (75.2)
Other race or multiracial^a^	610 566 (3.1)	172 666 (3.8)	137 384 (3.5)	221 422 (2.4)
Missing	433 097 (2.2)	107 902 (2.4)	91 853 (2.4)	170 163 (1.9)
Educational level, y				
≤8	639 732 (3.2)	287 457 (6.3)	111 767 (2.9)	39 140 (0.4)
9-11	1 849 106 (9.3)	950 166 (20.9)	348 283 (9.0)	110 365 (1.2)
12	4 847 467 (24.4)	1 853 022 (40.8)	1 319 218 (34.1)	922 015 (10.1)
≥13	12 535 688 (63.1)	1 453 572 (32.0)	2 093 742 (54.1)	8 045 761 (88.3)
Language (survey language)				
English	18 430 581 (91.9)	3 887 634 (84.6)	3 540 782 (90.7)	9 035 309 (98.5)
Spanish	1 564 765 (7.8)	682 363 (14.9)	349 954 (9.0)	132 250 (1.4)
Chinese	51 746 (0.3)	23 441 (0.5)	14 751 (0.4)	8825 (0.1)
Prepregnancy insurance status				
Uninsured	2 994 236 (15.0)	1 211 878 (26.4)	848 242 (21.7)	382 279 (4.2)
Public (Medicaid)	4 749 992 (23.7)	2 362 834 (51.5)	1 229 977 (31.5)	375 247 (4.1)
Private	12 269 301 (61.3)	1 010 457 (22.0)	1 822 783 (46.7)	8 415 843 (91.7)
Prenatal care (Kessner Index)				
Adequate	13 823 331 (69.0)	2 606 071 (56.7)	2 608 556 (66.8)	7 206 371 (78.5)
Intermediate	3 790 907 (18.9)	1 208 723 (26.3)	819 275 (21.0)	1 179 584 (12.9)
Inadequate	1 130 238 (5.6)	445 136 (9.7)	222 129 (5.7)	248 232 (2.7)
Unknown	1 302 617 (6.5)	333 508 (7.3)	255 528 (6.5)	542 197 (5.9)
Pregnancy-related conditions				
Diabetes (preexisting or gestational)	2 348 105 (11.7)	601 286 (13.1)	491 930 (12.6)	994 190 (10.8)
Hypertension during pregnancy	1 904 416 (9.5)	456 960 (10.0)	396 511 (10.2)	846 235 (9.2)
History of preterm birth	652 175 (3.4)	216 593 (5.0)	146 579 (4.0)	213 813 (2.5)
Smoking during pregnancy				
No	17 167 741 (92.2)	3 496 389 (83.0)	3 280 539 (90.3)	8 359 438 (97.6)
Yes	1 446 648 (7.8)	717 334 (17.0)	352 713 (9.7)	208 883 (2.4)
Infant gestational age, wk				
≤27	111 837 (0.6)	31 826 (0.7)	24 732 (0.6)	40 487 (0.4)
28-33	365 290 (1.8)	102 919 (2.2)	75 010 (1.9)	137 636 (1.5)
34-36	1 269 350 (6.3)	342 138 (7.5)	247 004 (6.3)	517 205 (5.6)
37-42	18 258 735 (91.1)	4 104 796 (89.4)	3 549 953 (90.9)	8 466 567 (92.3)
≥43	4322 (0.02)	856 (0.02)	630 (0.02)	1543 (0.02)
Missing	37 560 (0.2)	10 902 (0.2)	8158 (0.2)	12 947 (0.1)
Infant plurality				
Singleton	19 628 479 (98.2)	4 515 238 (98.6)	3 836 139 (98.5)	8 947 145 (97.9)
Multiple	356 172 (1.8)	66 043 (1.4)	56 854 (1.5)	196 382 (2.2)

Abbreviations: FPL, federal poverty level; PRAMS, Pregnancy Risk Assessment Monitoring System.

^a^ Specific races and ethnicities not specified in the PRAMS dataset.

**Figure 1.**
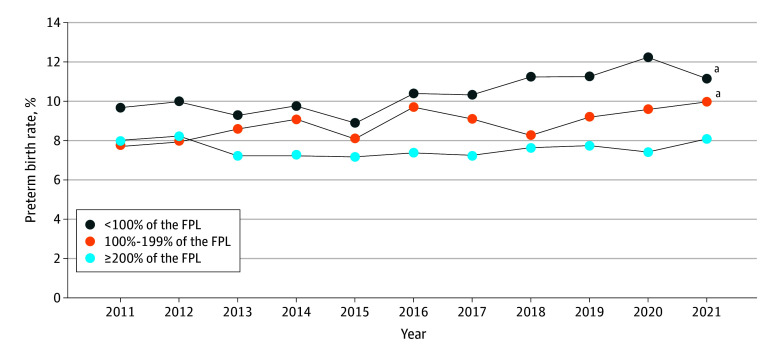
Trends of Preterm Birth by Household Income in the US, 2011-2021 FPL indicates federal poverty level. ^a^*P* < .001.

**Figure 2.**
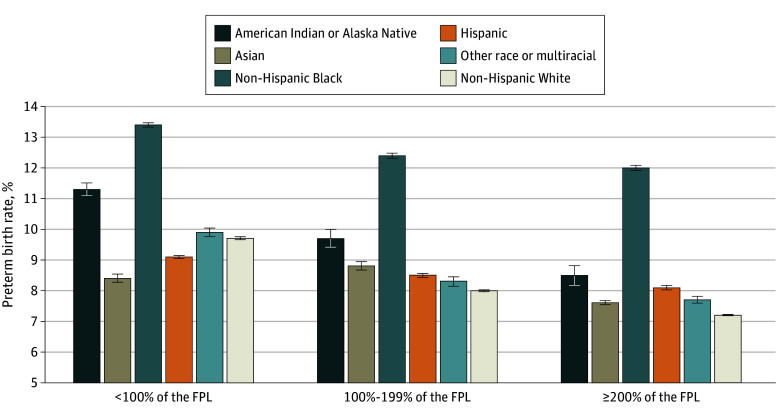
Preterm Birth Rates by Race and Ethnicity and Income Error bars indicate 95% CIs. FPL indicates federal poverty level.

From 2011 to 2021, preterm birth rates increased in the group with incomes less than 100% of the FPL (2011, 9.7%; 2021, 11.1%; *P* < .001) and in the gorup with incomes 100% to 199% of the FPL (2011, 7.8%; 2021, 10.0%; *P* < .001), but not in the group with incomes 200% or more of the FPL (2011, 8.0%; 2021, 8.1%; *P* = .15) (Figure 1). Preterm birth rates were highest for households living below the poverty line (<100% of the FPL) for all racial and ethnic groups, except Asian. Black mothers had the highest rates of preterm birth compared with other racial and ethnic groups across all household income levels (Figure 2). The association of income with preterm birth remained significant after adjusting for sociodemographic and pregnancy-related covariates, but attenuated to the null after including race and ethnicity in the model (Table 2). Interaction models showed that in the lowest income group, Black mothers had a 19% greater risk of preterm birth compared with White mothers (adjusted relative risk [ARR], 1.19; 95% CI, 1.11-1.27), whereas in the highest income group, Black mothers had a 13% greater risk of preterm birth compared with White mothers (ARR, 1.13; 95% CI, 1.01-1.26) (Table 3). Among White mothers, those in the highest income group had a 9% lower risk of preterm birth compared with their counterparts with low income (ARR, 0.91; 95% CI, 0.85-0.99). Sensitivity analyses including the “missing income” category and using multiple imputation for missing income yielded similar results (eTables 2-6 in Supplement 1).

**Table 2.** Association of Household Income and Preterm Birth Among US Mothers (PRAMS 2011-2021)

Household income	Model 1^a^		Model 2^b^		Model 3^c^	
	RR (95% CI)	*P* value	RR (95% CI)	*P* value	RR (95% CI)	*P* value
<100% of the FPL	1.00 [Reference]	NA	1.00 [Reference]	NA	1.00 [Reference]	NA
100%-199% of the FPL	0.85 (0.82-0.88)	<.001	0.98 (0.94-1.03)	.41	1.00 (0.96-1.04)	.99
≥200% of the FPL	0.73 (0.71-0.75)	<.001	0.93 (0.88-0.97)	.003	0.97 (0.93-1.03)	.43

Abbreviations: FPL, federal poverty level; NA, not applicable; PRAMS, Pregnancy Risk Assessment Monitoring System; RR, relative risk.

^a^ Unadjusted association of income and preterm birth.

^b^ Adjusted for maternal age, maternal educational level, maternal language, maternal prepregnancy insurance status, maternal diabetes prior to or during pregnancy, maternal hypertension during pregnancy, history of previous preterm birth, infant plurality, prenatal care, and smoking during pregnancy.

^c^ Model 2 adjusted additionally for maternal race and ethnicity.

**Table 3.** Adjusted RR of Preterm Birth Across Racial and Ethnic Groups Within Income Categories

Income level and race and ethnicity	Adjusted RR (95% CI)	*P* value
**<100% of the FPL**		
American Indian or Alaska Native	0.95 (0.83-1.10)	.50
Asian	0.95 (0.81-1.12)	.56
Hispanic	0.98 (0.90-1.07)	.69
Non-Hispanic Black	1.19 (1.11-1.27)	<.001
Non-Hispanic White	1.00 [Reference]	NA
Other race or multiracial	0.97 (0.84-1.12)	.65
**100%-199% of the FPL**		
American Indian or Alaska Native	0.95 (0.75-1.20)	.93
Asian	1.10 (0.90-1.37)	.18
Hispanic	0.99 (0.89-1.11)	.50
Non-Hispanic Black	1.03 (0.93-1.13)	.17
Non-Hispanic White	0.96 (0.89-1.02)	.26
Other race or multiracial	0.98 (0.78-1.22)	.85
**≥200% of the FPL**		
American Indian or Alaska Native	1.09 (0.86-1.37)	.17
Asian	0.98 (0.82-1.18)	.51
Hispanic	0.97 (0.86-1.09)	.42
Non-Hispanic Black	1.13 (1.01-1.26)	<.001
Non-Hispanic White	0.91 (0.85-0.99)	.03
Other race or multiracial	0.94 (0.78-1.13)	.86

Abbreviations: FPL, federal poverty level; NA, not applicable; RR, relative risk.

## Discussion

We found an inverse association between household income and preterm birth, with the highest rates among families living below the poverty line and the lowest among those at or above 200% of the FPL. Our study builds on the existing literature on the association of socioeconomic factors with birth outcomes by examining temporal trends at a national level. Our finding of increasing preterm birth rates in the lowest income categories coupled with stable rates in the highest income category underscores growing income-based disparities that warrant further investigation.

We found a persistent and significant disparity in preterm birth rates experienced by Black mothers across all income levels. Black mothers in the highest income group experienced higher rates of preterm birth than White mothers in the lowest income group. Consistent with recent literature, our interaction analysis revealed that income may serve as a protective factor against preterm birth but that the magnitude of this protective effect differs across racial and ethnic groups.^12,13^ Factors beyond income, such as structural racism and discrimination and associated health-related social needs, chronic stress, and differential access to quality care, are likely associated with the persistently elevated risk of poor gestational outcomes among Black women.^14,15,16^

This study has implications for public health efforts aimed at advancing maternal and child health through reducing preterm birth rates. Interventions focused solely on addressing income disparities may be insufficient to eliminate the racial and ethnic disparities in preterm birth observed. A multifaceted approach is needed that addresses both socioeconomic factors and the underlying structural inequities that disproportionally affect Black communities. This approach may include policies and interventions aimed at improving access to timely and comprehensive prenatal care, addressing maternal chronic health conditions and unmet social needs, reducing stress, and mitigating racial and ethnic discrimination in health care.^17^ Furthermore, our findings underscore that nationally representative and annually collected data such as PRAMS are critical to track how social factors may be associated with birth outcomes and to identify areas for targeted intervention.

### Limitations

Several limitations should be considered when interpreting our results. First, household income was self-reported in categorical ranges, which may introduce misclassification. However, we used a conservative approach to estimate income status based on published methods. Second, our study used cross-sectional data, which limits our ability to make causal inference. Third, although we adjusted for several important sociodemographic and pregnancy-related factors, there may be other unmeasured factors that could influence the observed associations.

## Conclusions

In this population-based, cross-sectional study of more than 400 000 US mother-infant dyads from 2011 to 2021, disparities in preterm birth rates by household income widened over time, with the highest rates observed among families living below the FPL. Black mothers remained at significantly higher risk of preterm birth compared with White mothers in both the lowest and highest income groups, suggesting that racial and ethnic disparities were not fully explained by income. These findings underscore the need to identify and address the structural and modifiable pathways through which poverty and racism are jointly associated with disparities in preterm birth rates.

## Notes

Supplement 1. **eTable 1.** Comparison of Populations With Complete vs Missing Income Data **eTable 2.** Sensitivity Analyses Adding a ‘Missing Income’ Category: Baseline Characteristics of Mother-Infant Dyads in the PRAMS Study Population, 2011–2021 (N = 412,133; Weighted 20 Million) **eTable 3.** Sensitivity Analyses Adding a ‘Missing Income’ Category: Association of Household Income and Preterm Birth Among U.S. Mothers (PRAMS, 2011–2021) **eTable 4.** Sensitivity Analyses Adding a ‘Missing Income’ Category: Adjusted Relative Risk of Preterm Birth Across Racial and Ethnic Groups Within and Across Income Categories **eTable 5.** Sensitivity Analyses Using Multiple Imputation for Missing Income: Association of Household Income and Preterm Birth Among U.S. Mothers (PRAMS, 2011–2021) **eTable 6.** Sensitivity Analyses Using Multiple Imputation for Missing Income: Adjusted Relative Risk of Preterm Birth Across Racial and Ethnic Groups Within and Across Income Categories

Supplement 2.
Data Sharing Statement
