# Supplementary material for: Trends in US Preterm Birth Rates by Household Income and Race and Ethnicity
Source: JAMA Netw Open. 2026 Jan 2;9(1):e2550664. doi: 10.1001/jamanetworkopen.2025.50664 (PMC12761331; doi:10.1001/jamanetworkopen.2025.50664)
Supplement: Supplement 1. — eTable 1. Comparison of Populations With Complete vs Missing Income Data eTable 2. Sensitivity Analyses Adding a ‘Missing Income’ Category: Baseline Characteristics of Mother-Infant Dyads in the PRAMS Study Population, 2011–2021 (N = 412,133; Weighted 20 Million) eTable 3. Sensitivity Analyses Adding a ‘Missing Income’ Category: Association of Household Income and Preterm Birth Among U.S. Mothers (PRAMS, 2011–2021) eTable 4. Sensitivity Analyses Adding a ‘Missing Income’ Category: Adjusted Relative Risk of Preterm Birth Across Racial and Ethnic Groups Within and Across Income Categories eTable 5. Sensitivity Analyses Using Multiple Imputation for Missing Income: Association of Household Income and Preterm Birth Among U.S. Mothers (PRAMS, 2011–2021) eTable 6. Sensitivity Analyses Using Multiple Imputation for Missing Income: Adjusted Relative Risk of Preterm Birth Across Racial and Ethnic Groups Within and Across Income Categories [file jamanetwopen-e2550664-s001.pdf]

Supplementary Online Content

Cordova-Ramos EG, Ruiz SY, Guyol GG, et al. Trends in US preterm birth rates by household income and race and ethnicity. *JAMA Netw Open.* 2026;9(1):e2550664. doi:10.1001/jamanetworkopen.2025.50664

eTable 1. Comparison of Populations With Complete vs Missing Income Data
eTable 2. Sensitivity Analyses Adding a 'Missing Income' Category: Baseline Characteristics of Mother-Infant Dyads in the PRAMS Study Population, 2011–2021 (N = 412,133; Weighted 20 Million)
eTable 3. Sensitivity Analyses Adding a 'Missing Income' Category: Association of Household Income and Preterm Birth Among U.S. Mothers (PRAMS, 2011–2021)
eTable 4. Sensitivity Analyses Adding a 'Missing Income' Category: Adjusted Relative Risk of Preterm Birth Across Racial and Ethnic Groups Within and Across Income Categories
eTable 5. Sensitivity Analyses Using Multiple Imputation for Missing Income: Association of Household Income and Preterm Birth Among U.S. Mothers (PRAMS, 2011–2021)
eTable 6. Sensitivity Analyses Using Multiple Imputation for Missing Income: Adjusted Relative Risk of Preterm Birth Across Racial and Ethnic Groups Within and Across Income Categories

This supplementary material has been provided by the authors to give readers additional information about their work.

© 2026 Cordova-Ramos EG et al. *JAMA Network Open.*

eTable 1. Comparison of Populations With Complete vs Missing Income Data

Characteristic	Total	Missing income data (n=2,371,782)	With income data (n=17,675,310)	P-value*
Maternal age (years)				<0.001
≤19	1,011,263 (5.04)	298,098 (12.57)	713,165 (4.03)	
20–34	15,505,816 (77.35)	1,739,859 (73.36)	13,765,957 (77.88)	
≥35	3,529,608 (17.61)	333,776 (14.07)	3,195,833 (18.08)	
Race and ethnicity				<0.001
Non-Hispanic White	11,797,803 (60.15)	958,881 (41.54)	10,838,922 (62.63)	
Non-Hispanic Black	2,830,208 (14.43)	509,964 (22.09)	2,320,244 (13.41)	
Hispanic	3,108,217 (15.85)	598,464 (25.92)	2,509,752 (14.50)	
Asian	1,100,827 (5.61)	143,011 (6.19)	957,816 (5.53)	
American Indian/Alaska Native	166,355 (0.85)	19,189 (0.83)	147,166 (0.85)	
Other non-White/Multiracial	610,566 (3.11)	79,094 (3.43)	531,471 (3.07)	
Education (years)				<0.001
0-8	639,732 (3.22)	201,368 (8.61)	438,364 (2.50)	
9-11	1,849,106 (9.31)	440,293 (18.84)	1,408,813 (8.03)	
12	4,847,467 (24.39)	753,213 (32.22)	4,094,254 (23.35)	
≥13	12,535,688 (63.08)	942,613 (40.33)	11,593,075 (66.12)	
Language				<0.001
English	18,430,581 (91.94)	1,966,856 (82.93)	16,463,725 (93.15)	
Spanish	1,564,765 (7.81)	400,197 (16.87)	1,164,568 (6.59)	
Chinese	51,746 (0.26)	4,729 (0.20)	47,017 (0.27)	
Pre-pregnancy insurance status				<0.001
Uninsured	2,994,236 (14.96)	551,836 (23.44)	2,442,400 (13.83)	
Public (Medicaid)	4,749,992 (23.73)	781,934 (33.22)	3,968,059 (22.47)	
Private	12,269,301 (61.31)	1,020,217 (43.34)	11,249,083 (63.70)	
Infant gestational age				<0.001

© 2026 Cordova-Ramos EG et al. *JAMA Network Open.*

≤27	111,837 (0.56)	14,791 (0.63)	97,045 (0.55)	
28-33	365,290 (1.83)	49,725 (2.10)	315,565 (1.79)	
34-36	1,269,350 (6.34)	163,003 (6.89)	1,106,347 (6.27)	
37-42	18,258,735 (91.25)	2,137,418 (90.33)	16,121,317 (91.37)	
>43	4,322 (0.02)	1,293 (0.05)	3,029 (0.02)	

*Comparing population with complete vs. missing income data

© 2026 Cordova-Ramos EG et al. *JAMA Network Open.*

eTable 2. Sensitivity Analyses Adding a 'Missing Income' Category: Baseline Characteristics of Mother-Infant Dyads in the PRAMS Study Population, 2011–2021 (N = 412,133; Weighted 20 Million)

Characteristic	Total	<100% FPL n=4,593,438	100–199% FPL n=3,905,488	≥200% FPL n=9,176,385	Missing Income n=2,371,782
Maternal age, y					
≤19	1,011,263 (5.04)	485,796 (10.58)	173,902 (4.45)	53,467 (0.58)	298,098 (12.57)
20–34	15,505,816 (77.35)	3,600,038 (78.37)	3,202,526 (82.0)	6,963,393 (75.89)	1,739,859 (73.36)
≥35	3,529,608 (17.61)	507,526 (11.05)	528,961 (13.54)	2,159,346 (23.53)	333,776 (14.07)
Race and ethnicity					
Non-Hispanic White	11,797,803 (58.85)	1,894,702 (41.25)	2,039,967 (52.23)	6,904,253 (75.24)	958,881 (40.43)
Non-Hispanic Black	2,830,208 (14.12)	1,067,206 (23.23)	666,554 (17.07)	586,484 (6.39)	509,964 (21.50)
Hispanic	3,108,217 (15.50)	1,107,133 (24.10)	750,334 (19.21)	652,286 (7.11)	598,464 (25.23)
Asian	1,100,827 (5.49)	166,285 (3.62)	180,282 (4.62)	611,249 (6.66)	143,011 (6.03)
American Indian/Alaska Native	166,355 (0.83)	77,544 (1.69)	39,115 (1.0)	30,507 (0.33)	19,189 (0.81)
Other non-White/Multiracial	610,566 (3.05)	172,666 (3.76)	137,384 (3.52)	221,422 (2.41)	79,094 (3.33)
Missing	433,097 (2.16)	107,902 (2.35)	91,853 (2.35)	170,163 (1.85)	63,179 (2.66)
Education (years)					
0-8	639,732 (3.22)	287,457 (6.33)	111,767 (2.89)	39,140 (0.43)	201,368 (8.61)
9-11	1,849,106 (9.31)	950,166 (20.91)	348,283 (8.99)	110,365 (1.21)	440,293 (18.84)
12	4,847,467 (24.39)	1,853,022 (40.78)	1,319,218 (34.06)	922,015 (10.11)	753,213 (32.22)
≥13	12,535,688 (63.08)	1,453,572 (31.99)	2,093,742 (54.06)	8,045,761 (88.25)	942,613 (40.33)
Language					
English	18,430,581 (91.94)	3,887,634 (84.63)	3,540,782 (90.66)	9,035,309 (98.46)	1,966,856 (82.93)
Spanish	1,564,765 (7.81)	682,363 (14.86)	349,954 (8.96)	132,250 (1.44)	400,197 (16.87)
Chinese	51,746 (0.26)	23,441 (0.51)	14,751 (0.38)	8,825 (0.10)	4,729 (0.20)
Pre-pregnancy insurance status					
Uninsured	2,994,236 (14.96)	1,211,878 (26.43)	848,242 (21.74)	382,279 (4.17)	551,836 (23.44)
Public (Medicaid)	4,749,992 (23.73)	2,362,834 (51.53)	1,229,977 (31.53)	375,247 (4.09)	781,934 (33.22)
Private	12,269,301 (61.31)	1,010,457 (22.04)	1,822,783 (46.73)	8,415,843 (91.74)	1,020,217 (43.34)

© 2026 Cordova-Ramos EG et al. *JAMA Network Open.*

Prenatal care (Kessner Index)					
Adequate	13,823,331 (68.95)	2,606,071 (56.73)	2,608,556 (66.79)	7,206,371 (78.53)	1,402,332 (59.13)
Intermediate	3,790,907 (18.91)	1,208,723 (26.31)	819,275 (20.98)	1,179,584 (12.85)	583,325 (24.59)
Inadequate	1,130,238 (5.64)	445,136 (9.69)	222,129 (5.69)	248,232 (2.71)	214,742 (9.05)
Unknown	1,302,617 (6.50)	333,508 (7.26)	255,528 (6.54)	542,197 (5.91)	171,384 (7.23)
Pregnancy-related conditions					
Diabetes (pre-existing or gestational)	2,348,105 (11.71)	601,286 (13.09)	491,930 (12.60)	994,190 (10.83)	260,699 (10.99)
Hypertension during pregnancy	1,904,416 (9.50)	456,960 (9.95)	396,511 (10.15)	846,235 (9.22)	204,710 (8.63)
History of preterm birth	652,175 (3.44)	216,593 (5.01)	146,579 (3.96)	213,813 (2.45)	75,191 (3.36)
Smoking during pregnancy					
No	17,167,741 (92.23)	3,496,389 (82.98)	3,280,539 (90.29)	8,359,438 (97.56)	2,031,375 (92.37)
Yes	1,446,648 (7.77)	717,334 (17.02)	352,713 (9.71)	208,883 (2.44)	167,718 (7.63)
Infant gestational age					
≤27	111,837 (0.56)	31,826 (0.69)	24,732 (0.63)	40,487 (0.44)	14,791 (0.62)
28-33	365,290 (1.82)	102,919 (2.24)	75,010 (1.92)	137,636 (1.50)	49,725 (2.10)
34-36	1,269,350 (6.33)	342,138 (7.45)	247,004 (6.32)	517,205 (5.64)	163,003 (6.87)
37-42	18,258,735 (91.08)	4,104,796 (89.36)	3,549,953 (90.90)	8,466,567 (92.26)	2,137,418 (90.12)
>43	4,322 (0.02)	856 (0.02)	630 (0.02)	1,543 (0.02)	1,293 (0.06)
Missing	37,560 (0.19)	10,902 (0.24)	8,158 (0.21)	12,947 (0.14)	5,552 (0.23)
Infant Plurality					
Singleton	19,628,479 (98.22)	4,515,238 (98.56)	3,836,139 (98.54)	8,947,145 (97.85)	2,329,957 (98.44)
Multiple	356,172 (1.78)	66,043 (1.44)	56,854 (1.46)	196,382 (2.15)	36,893 (1.56)

© 2026 Cordova-Ramos EG et al. *JAMA Network Open.*

eTable 3. Sensitivity Analyses Adding a 'Missing Income' Category: Association of Household Income and Preterm Birth Among U.S. Mothers (PRAMS, 2011–2021)

	Model 1*		Model 2**		Model 3***	
	RR (95% CI)	P-value	RR (95% CI)	P-value	RR (95% CI)	P-value
Household income						
<100% FPL	Reference					
100 – 199% FPL	**0.85 (0.82-0.89)**	**<0.001**	0.98 (0.94-1.02)	0.305	0.99 (0.95-1.04)	0.797
≥200% FPL	**0.73 (0.71-0.75)**	**<0.001**	**0.93 (0.88-0.96)**	**<0.001**	0.97 (0.92-1.01)	0.137
Missing	**0.92 (0.88-0.96)**	**<0.001**	0.97 (0.93-1.03)	0.354	0.98 (0.93-1.02)	0.322

FPL: Federal Poverty Level

(*) Model 1: Unadjusted association of income and preterm birth.

(**) Model 2: Adjusting for maternal age, maternal education, maternal language, maternal pre-pregnancy insurance status, maternal diabetes prior or during pregnancy, maternal hypertension during pregnancy, history of previous preterm birth, infant plurality, prenatal care, and smoking during pregnancy.

(***) Model 3: Model 2 adjusting additionally for maternal race and ethnicity.

© 2026 Cordova-Ramos EG et al. *JAMA Network Open.*

eTable 4. Sensitivity Analyses Adding a 'Missing Income' Category: Adjusted Relative Risk of Preterm Birth Across Racial and Ethnic Groups Within and Across Income Categories

Income Level	Race/Ethnicity	Adjusted RR (95% CI)	P-value
<100% FPL	Non-Hispanic White (ref)	1.00	
	Non-Hispanic Black	**1.19 (1.11-1.27)**	**<0.001**
	Hispanic	0.96 (0.89-1.05)	0.463
	Asian	0.94 (0.80-1.11)	0.516
	American Indian/Alaska Native	0.95 (0.83-1.10)	0.547
	Other non-White/Multiracial	0.96 (0.83-1.12)	0.680
100-199% FPL	Non-Hispanic White	0.95 (0.89-1.02)	0.190
	Non-Hispanic Black	1.02 (0.92-1.13)	0.166
	Hispanic	0.99 (0.88-1.11)	0.443
	Asian	1.10 (0.89-1.37)	0.166
	American Indian/Alaska Native	0.94 (0.74-1.19)	0.925
	Other non-White/Multiracial	0.97 (0.78-1.21)	0.857
≥200% FPL	Non-Hispanic White	**0.92 (0.86-0.97)**	**0.007**
	Non-Hispanic Black	**1.12 (1.02-1.23)**	**<0.001**
	Hispanic	0.97 (0.86-1.09)	0.311
	Asian	0.98 (0.82-1.17)	0.473
	American Indian/Alaska Native	1.08 (0.86-1.35)	0.169
	Other non-White/Multiracial	0.93 (0.77-1.12)	0.865
Missing/Unknown	Non-Hispanic White	0.94 (0.86-1.02)	0.155
	Non-Hispanic Black	0.99 (0.88-1.13)	0.290
	Hispanic	0.98 (0.86-1.12)	0.504
	Asian	1.05 (0.83-1.34)	0.339
	American Indian/Alaska Native	1.02 (0.77-1.34)	0.576
	Other non-White/Multiracial	0.95 (0.73-1.24)	0.897

© 2026 Cordova-Ramos EG et al. *JAMA Network Open.*

eTable 5. Sensitivity Analyses Using Multiple Imputation for Missing Income: Association of Household Income and Preterm Birth Among U.S. Mothers (PRAMS, 2011–2021)

	**Model 1***		**Model 2****		**Model 3*****	
	**RR (95% CI)**	**P-value**	**RR (95% CI)**	**P-value**	**RR (95% CI)**	**P-value**
Household income						
<100% FPL	Reference					
100 – 199% FPL	**0.86 (0.83-0.90)**	**<0.001**	0.98 (0.94-1.01)	0.300	0.99 (0.95-1.03)	0.793
≥200% FPL	**0.73 (0.71-0.76)**	**<0.001**	**0.92 (0.88-0.96)**	**0.001**	0.97 (0.93-1.02)	0.247

FPL: Federal Poverty Level
(*) Model 1: Unadjusted association of income and preterm birth.
(**) Model 2: Adjusting for maternal age, maternal education, maternal language, maternal pre-pregnancy insurance status, maternal diabetes prior or during pregnancy, maternal hypertension during pregnancy, history of previous preterm birth, infant plurality, prenatal care, and smoking during pregnancy.
(***) Model 3: Model 2 adjusting additionally for maternal race and ethnicity.

© 2026 Cordova-Ramos EG et al. *JAMA Network Open.*

eTable 6. Sensitivity Analyses Using Multiple Imputation for Missing Income: Adjusted Relative Risk of Preterm Birth Across Racial and Ethnic Groups Within and Across Income Categories

Income Level	Race/Ethnicity	Adjusted RR (95% CI)	P-value
<100% FPL	Non-Hispanic White (ref)	1.00	
	Non-Hispanic Black	**1.19 (1.12-1.28)**	**<0.001**
	Hispanic	0.97 (0.89-1.05)	0.474
	Asian	0.94 (0.80-1.10)	0.458
	American Indian/Alaska Native	0.96 (0.84-1.09)	0.538
	Other non-White/Multiracial	0.98 (0.85-1.12)	0.762
100-199% FPL	Non-Hispanic White	0.96 (0.89-1.03)	0.241
	Non-Hispanic Black	1.02 (0.92-1.12)	0.236
	Hispanic	0.99 (0.89-1.11)	0.524
	Asian	1.10 (0.89-1.35)	0.192
	American Indian/Alaska Native	0.96 (0.76-1.21)	0.998
	Other non-White/Multiracial	0.96 (0.78-1.19)	0.990
≥200% FPL	Non-Hispanic White	**0.92 (0.87-0.98)**	**0.010**
	Non-Hispanic Black	**1.19 (1.01-1.21)**	**<0.001**
	Hispanic	0.97 (0.87-1.09)	0.273
	Asian	1.00 (0.84-1.19)	0.323
	American Indian/Alaska Native	1.09 (0.86-1.36)	0.142
	Other non-White/Multiracial	0.92 (0.76-1.10)	0.991

© 2026 Cordova-Ramos EG et al. *JAMA Network Open.*
