# Supplementary material for: Trends in US Preterm Birth Rates by Household Income and Race and Ethnicity
Source: JAMA Netw Open. 2026 Jan 2;9(1):e2550664. doi: 10.1001/jamanetworkopen.2025.50664 (PMC12761331; doi:10.1001/jamanetworkopen.2025.50664)
Supplement: Supplement 2. — Data Sharing Statement [file jamanetwopen-e2550664-s002.pdf]

Data Sharing Statement

Cordova-Ramos. Trends in US Preterm Birth Rates by Household Income and Race and Ethnicity. *JAMA Netw Open*. Published January 02, 2026.
doi:10.1001/jamanetworkopen.2025.50664

Data
Data available: Yes
Data types: Deidentified participant data
How to access data: Data are available upon request to the CDC Pregnancy Risk Assessment Monitoring System Working Group.
When available: With publication

Supporting Documents
Document types: None

Additional Information
Who can access the data: Researchers whose proposed use of the data has been approved
Types of analyses: For any approved purposes
Mechanisms of data availability: After approval by the CDC Pregnancy Risk Assessment Monitoring System Working Group
